# Risk Factors and Complications Among Pediatric Patients With Sickle Cell Anemia: A Single Tertiary Center Retrospective Study

**DOI:** 10.7759/cureus.12440

**Published:** 2021-01-03

**Authors:** Fatma Alzahrani, Anas M Fallatah, Fatimah M Al-Haddad, Shahad T Khayyat, Wasayf M AlMehmadi, Bashaier G AlQahtani, Rawabi S Alamri

**Affiliations:** 1 Pediatrics, King Abdulaziz University Hospital, Jeddah, SAU; 2 Medicine, College of Medicine, King Abdulaziz University, Jeddah, SAU; 3 Medicine, College of Medicine, Imam Abdulrahman Bin Faisal University, Dammam, SAU; 4 Medicine, College of Medicine, Taif University, Taif, SAU

**Keywords:** sickle cell anemia, sickle cell disease, risk factors, sickle cell complications, jeddah, saudi arabia.

## Abstract

Background and objective

Sickle cell anemia (SCA) is one of the common genetic diseases in the Kingdom of Saudi Arabia (KSA). This disease results from a genetic mutation that causes malformation of the red blood cells (RBCs), leading to various systemic complications, including vaso-occlusive crisis (VOC), acute chest syndrome (ACS), osteomyelitis, avascular necrosis (AVN), and stroke, to name a few. The leading cause of mortality in SCA is these systemic complications rather than the disease itself. Understanding the risk factors of these complications can help reduce mortality in these patients and improve their quality of life. In this study, we aimed to determine the risk factors of SCA complications among pediatric patients with SCA at King Abdulaziz University Hospital (KAUH) in Jeddah, KSA.

Methods

This retrospective study was carried out from January 2012 till June end 2019. It was conducted among pediatric patients with SCA. Patients were screened for eligibility, and we excluded those with thalassemia and those who had a medical history of chronic diseases. Data were collected from patients’ electronic medical records.

Results

The study included 102 pediatric patients with SCA; their mean age was 7.88 ±4.22 years; almost half of them were females (56%) and 44% were males. The dominant body mass index (BMI) classification among them was normal (49%). Urinary tract infection (UTI) was the most common complication with 38 cases followed by VOC with 32 cases. Other complications observed were ACS (25.5%) followed by stroke (15.7%). HbSS was the most prominent genotype among these patients, and it was associated with a higher rate of complications. However, there was no significant relationship between genotype and patients developing complications. Finally, patients with high white blood cell (WBC) counts, elevated systolic blood pressure (SBP), and hypoxia developed more complications, and there was a significant relationship between these conditions and the development of complications (p<0.05).

Conclusion

Based on our findings, patients with high WBC count, elevated SBP, and hypoxia are at greater risk of developing complications. Accordingly, healthcare providers should consider putting in place all measures required to provide a good quality of life for these patients, including raising awareness about the risk factors that lead to these complications, appropriate immunizations, and precautionary measures to promote these patients' welfare.

## Introduction

Sickle cell disease (SCD) is an increasingly universal health issue. It is a hereditary form of anemia, which is caused by a single point mutation in the beta-globin chain of hemoglobin (Hb) that is a substitution of amino acid glutamate into valine at position number six along the chain. This mutation leads to the production of sickle Hb (HbS), which is less soluble than normal fetal or adult Hb. SCD refers to a group of syndromes in which sickle mutation is co-inherited with another mutation found in the other beta chain; sickle cell anemia (SCA) is one of them, which is defined by homozygosity of sickle mutation. It is characterized by the presence of sickle-shaped erythrocytes [1].

Hemoglobinopathy is the most common autosomal recessive disease, with 350,000 newborns affected per year [2]. SCD was first reported in the Kingdom of Saudi Arabia (KSA) in the 1960s [3]. It led to the introduction of nationwide screening methods to establish the disease clinical spectrum and genotype variations throughout KSA [4]. A study conducted in Makkah found that 108 patients were diagnosed with SCD out of 45,682 young adults and children living in the western region of KSA [5]. Regarding the regional distribution of SCD in KSA, the highest prevalence has been found in the Eastern Province, followed by the western and the central regions; no cases were found in the northern region [5].

SCA is a multi-organ disorder with wide variability in clinical presentations and severity among anemic children. Major clinical features are related to chronic hemolytic anemia, infections, or vaso-occlusive crisis (VOC) [1]. Complications related to SCA are associated with high rates of morbidity and mortality. They can be categorized into two main types: acute and chronic. Acute complications are vaso-occlusive, hemolytic, and splenic sequestration crises. In contrast, chronic complications are divided into two different categories based on their pathological cause [1]. One set is related to large vessel vasculopathy, such as cerebrovascular disease, pulmonary hypertension (PHTN), priapism, and retinopathy. The other set is due to the ongoing ischemic organ damage like hyposplenism, renal failure, bone disease, and liver damage [1].

These complications have been found to lead to frequent ER visits, re-admissions, prolonged hospital stays, and higher mortality rates [6]. According to recent literature, these complications are associated with certain factors such as elevated white blood cell (WBC) counts, low levels of fetal Hb (HbF), high body mass index (BMI) ratio, and high blood pressure [7].

To the best of our knowledge, there have been no recent local studies in KSA investigating the risk factors associated with SCD complications. Hence, in this study, we aimed to identify the complications of SCD as well as the related risk factors among pediatric patients with SCA at King Abdulaziz University Hospital (KAUH) in Jeddah, KSA.

## Materials and methods

This retrospective record review study was conducted among pediatric patients with SCA at KAUH, Jeddah, KSA. The study was carried out from January 2012 to June 2019. The patients’ data were obtained from their electronic medical records from the Medical Report Department in KAUH. The study was approved by the Institutional Review Board and the Research Ethics Committee of KAUH. A total of 102 patients were included in our study. Patients diagnosed with other types of hematological disorders (such as thalassemia or glucose 6 phosphate dehydrogenase deficiency) and those with a medical history of chronic diseases were excluded.

After enrolling the eligible participants, we developed a well-designed, structured electronic data sheet that included the following to collect patient data: demographic information (nationality, gender, age, weight, height, and BMI). BMI was defined for age using the World Health Organization (WHO) guidelines for children less than five years of age and those who were 5-19 years of age. Patients with a BMI below -2 standard deviations (SDs) were considered the underweight class, those above -2 SDs and below +1 SDs were categorized as normal, those above +1 SDs and below +2 SDs were regarded as overweight, and those with a BMI above +2 SDs were defined as obese [8].

Laboratory data were obtained from the initial visits, and they included Hb levels, WBC count, total bilirubin, and direct bilirubin. The other variables taken into account were as follows: vital signs upon admission (blood pressure, temperature, oxygen saturation), SCA genotype (HbSS, HbSC), current use of hydroxyurea (HU) or folic acid, and vaccination for pneumococcal infection; a history of previous strokes and their frequency, frequency of previous acute chest syndrome (ACS), frequency of previous pain crisis, and history of previous VOC. Disease complications that were addressed in the study were avascular necrosis (AVN), gallstones, and PHTN. Infections such as osteomyelitis, meningitis, and urinary tract infection (UTI) were also recorded. Mortality was also investigated.

Data were revised for accuracy in Microsoft Excel for Mac version 16. All statistical analyses were performed using SPSS Statistics version 26 (IBM, Armonk, NY). Frequencies and percentages were calculated for categorical variables, and measures of central tendency were calculated for continuous variables. Statistical differences between groups defined by each demographic variable (e.g., disease genotype, nationality) were examined through the chi-squared test and independent t-test for two-group comparisons. A p-value of less than 0.05 was considered statistically significant.

## Results

This study aimed to analyze the complications of SCD and identify the related risk factors among SCD children at KAUH. In our study, 102 patients were included over the last seven years. Table 1 shows the demographic data of our sample. Their age ranged from six months to 16 years with a mean of 7.91 ±4.19 years. 

**Table 1:** Demographic data of the sample population SD: standard deviation; BMI: body mass index; SBP: systolic blood pressure; DBP: diastolic blood pressure; ACS: acute chest syndrome

Variables	Values
Age (years), mean ±SD	7.91 ±4.19
Height (m), mean ±SD	116.68 ±25.79
Weight (kg), mean ±SD	22.25 ±11.31
BMI (kg/m^2^), mean ±SD	18.19 ±26.74
Nationality	
Saudi, n (%)	55 (54%)
Non-Saudi, n (%)	47 (46%)
Sex	
Male, n (%)	45 (44%)
Female, n (%)	57 (56%)
SC genotype	
HbSS, n (%)	71 (69%)
HbSC, n (%)	31 (30%)
SBP (mmHg), mean ±SD	111.63 ±13.091
DBP (mmHg), mean ±SD	62.33 ±10.971
Total bilirubin, mean ±SD	53.39 ±65.97
Direct bilirubin, mean ±SD	19.38 ±52.89
Number of previous pain crises, mean ±SD	6.78 ±9.637
Number of previous ACS, mean ±SD	0.35 ±0.713
History of previous strokes, mean ±SD	0.20 ±0.581

Most of the sample population had average systolic blood pressure (SBP) of 112 mmHg; the highest value was 162 mmHg, while the lowest was 82 mmHg. Similarly, the majority had average diastolic blood pressure (DBP) of 62 mmHg; the highest value was 102 mmHg, while the lowest was 41 mmHg. The frequency of VOC was as follows: 31.4% (32) had episodes of VOC, while 68.6% (70) did not. Most of the sample (84.3%, 86) had no history of stroke. Regarding ACS attacks among our sample, the maximum number of attacks was only four. Only a small percentage (15.7%, 16) had faced stroke episodes. We were able to obtain the total bilirubin values for 91 patients, and it ranged from 7 to 507, with an average of 53.3. Similarly, direct bilirubin values were obtained for 82 patients, and it ranged from 2 to 415, with an average of 19.3.

Table 2 demonstrates the different risk factors among Saudi and Non-Saudi patients. Leukocytosis was higher in non-Saudi patients, with 31 cases. However, leukocytosis was found in 20% of Saudi patients. The majority of the sample were anemic (94.1%); moreover, more than half of them were non-Saudi, whereas hyperthermia developed in only 12.7% of the cases. None of the Saudi patients had developed hypoxia. Furthermore, hypoxia was only found in one of the non-Saudi patients. One-third of Saudi patients were underweight, whereas the majority of non-Saudi patients had normal BMI. The predominant SCD genotype in the sample was HbSS, with 69.6%; 35.3% of them were Saudis, and 34.3% were non-Saudis. HbSC genotype was found in 30.4%; 10.8% of them were Saudis, and 19.6% were non-Saudis. Of note, 89.2% of the patients were taking folic acid as part of their treatment, while 59.8% of the patients were taking HU, and 26.7% had taken the pneumococcal vaccine.

**Table 2:** Risk factors related to SCD complications among our sample SCD: sickle cell disease; WBC: white blood cells; BMI: body mass index

Variables	Risk factors		Frequency	P-value
	Saudis	Non-Saudis		
	(N=55)	(N=47)		
WBC count				
Leukocytopenia	5	2	7	0.001
Leukocytosis	20	31	51	
Hemoglobin class				
Anemia	44	52	96	-
Non-anemic	3	3	6	
Oxygen saturation				
Hypoxia	0	1	1	-
Temperature				
Hyperthermia	7	6	13	-
BMI class				
Underweight	18	17	35	-
Normal	21	29	50	
Overweight	4	2	6	
Obese	4	5	9	
SCD genotype				
HbSS	36	35	71	-
HbSC	11	20	31	
Hydroxyurea				
Yes	30	31	61	-
No	17	24	41	
Folic acid				
Yes	42	49	91	-
No	5	6	11	
Pneumococcal vaccination				
Yes	11	16	27	-
No	44	31	75	

Table 3 addresses the various complications among our patients. The majority of them developed VOC with equal prevalence in both groups of nationalities. Also, UTI was most prevalent among non-Saudis with a frequency of 26 cases. UTI incidence appeared to be statistically significant with foreign patients. Moreover, osteomyelitis also was frequently seen among them with 17 cases. Also, meningitis, stroke, and AVN were likewise more frequent among non-Saudis patients. AVN incidence also appeared to be statistically significant, with a p-value of 0.049. Furthermore, Saudi patients appeared to be more commonly affected with gallstones, with a 100% frequency among those with gallbladder diseases. Severe SCA complications such as PHTN and death were only seen among non-Saudis. Regarding the SCA genotype, HbSS had a high rate of complications such as CVA, VOC, and UTI. On the other hand, HbSC had lower rates of these adverse complications. There was a significant association between the degree of oxygen saturation and VOC with a p-value of 0.001. Conversely, there was no significant association between the levels of WBC or Hb and SCA complications. Elevated SBP had a significant association with adverse complications (p<0.001).

**Table 3:** Summary of complications among our sample

Variables	Complications		Frequency	P-value
	Saudis	Non-Saudis		
	(N=55)	(N=47)		
Vaso-occlusive crisis (VOC)				
Yes	16	16	32	0.001
Urinary tract infection (UTI)				
Yes	12	26	38	0.028
Osteomyelitis				
Yes	6	17	23	0.028
Meningitis				
Yes	2	5	7	-
Ischemic stroke				
Yes	5	11	16	-
Avascular necrosis (AVN)				
Yes	5	8	13	0.049
Gallstone				
Yes	4	0	4	-
Pulmonary hypertension (PHTN)				
Yes	0	1	1	-
Death				
Yes	0	1	1	-

The study also analyzed the relationship between ACS attacks and some factors that might affect the number of attacks, including age, BMI, oxygen saturation, Hb level, WBC count, SBP, and DBP. The results showed a positive relationship between the number of ACS attacks and the WBC level. As the WBC count increases, the number of ACS attacks will increase. This correlation was found to be statistically significant (p<0.001). Moreover, a positive correlation existed between the number of ACS and all other mentioned factors. However, these correlations were found to be statistically insignificant in which the p-value was >0.05. Similarly, the relationships between the factors mentioned above and the number of painful crises or strokes were also studied. All of them showed positive correlations but were statistically insignificant.

## Discussion

This study aimed to assess the complications of SCD and identify the related factors among pediatric patients. SCA represents a significant global healthcare burden and significantly contributes to patient morbidity and mortality. Over the last decade, SCD has been on the rise in KSA with a prevalence of five out of 1,000 for sickle cell traits, and that of 0.38 out of 1,000 for individuals affected with the disease, according to the Premarital Screening Program [9]. Moreover, the newborn screening test has estimated a prevalence of 21% for sickle cell traits and 2.6% for SCA among newborns [10]. Frequent emergency department (ED) visits are considered a risk factor for adverse patient outcomes [11]. According to a recent study, one out of five patients has three or more ED visits per year [12]. Most of the time, those patients with high ED visits are mistreated and stigmatized as opioid abusers [13].

In our study, there was no major difference regarding the prevalence of SCA in terms of gender. This finding has been consistent throughout the literature [5]. Although the study showed no significant difference with regard to nationality, a recent multicenter study from Makkah revealed that a higher number of patients with SCA in their sample were Saudi nationals [5]. This variation can be attributed to the differences in the sampling pool, as the latter had collected data from three different hospital settings.

Regarding the risk factors of SCD complications, WBCs were found to be involved in the pathogenesis; however, the mechanism has not been completely understood. WBCs have been proved to be involved in the occlusion of blood vessels. This occlusion is mainly seen in the arteries and is found to be associated with disease severity [14]. Nevertheless, the majority of our sample had elevated WBC counts; however, it was not statistically significant. Another risk factor is the BMI class, and in this study, the majority of the sample were underweight to normal-weight patients (85 cases). This data is consistent with that of previously published literature, suggesting that patients with SCD are more prone to be in these categories due to nutritional deficiencies [15]. High metabolic rates, reduced absorption, and increased degradation are believed to be the mechanism behind it [16]. However, recent studies have shown that increased BMI among patients with SCD is associated with elevated blood pressure and hypertension [17]. Elevated blood pressure among SCD patients is associated with kidney disease, stroke, and early mortality [17].

Currently, HU is the only therapeutic agent that has been found to alter the course of SCD and improve patients’ outcomes, and this has been established via multiple randomized clinical trials over the years. Despite the bone marrow toxicity, HU has been found to reduce the rate of VOC, ACS, RBC transfusion, hospital duration, and is associated with improved survival rates [18]. Complete blood count (CBC) monitoring is warranted in cases of HU use. Although 60% of this study sample used HU, further studies are needed to investigate the barriers to using it.

Since most of the patients with SCD suffer from functional asplenia in the first year of their life, encapsulated organisms such as *Streptococcus pneumoniae (S. pneumoniae)* are known to cause major infections among them [19]. A total of 27 patients (26.5%) in our sample had been vaccinated against pneumococcal infection. This low number can be attributed to different reasons, and underutilization of vaccination facilities could be one among them. The pneumococcal vaccine has been proven to reduce the incidence of invasive pneumococcal infection among the pediatric population [20]. 

Despite the fact that there were only 13 cases of documented fever on admission in our cohort, it can usually be seen among patients with SCD with infectious and non-infectious etiologies such as sickle cell crisis. One of the most frequent complications among our sample was UTI, with 38 cases. Pediatric patients with SCD are more prone to suffer from bacterial infection due to the early loss of splenic function [21]. UTI is a major cause of morbidity among those patients. A study conducted in Nigeria found that 60 cases developed UTI [21]. However, no significant association was found in both studies.

VOC was observed to be the second most prevalent complication (32 cases), with 16 cases among both groups of nationalities. A recent local study conducted in Makkah found that 81 cases had VOC, and most of them were Saudis [5]. Although a single case had hypoxia upon admission, it is known that hypoxia is one of the risk factors for VOC. Moreover, there was a significant association between the degree of oxygen saturation and VOC with a p-value of less than 0.05. Osteomyelitis and VOC can be identical in the acute stage of the clinical presentation [22]. Patients with SCA are susceptible to a rare form of osteomyelitis caused by *Salmonella* species [23]. Iron overload, tissue infarction, and functional asplenia can result in immunodeficiency, which puts them at a higher risk of developing it [24]. A total of 23 cases were found in our sample, which is higher compared to a recent multicenter study conducted in Jazan (22 cases), including pediatrics and adult patients with SCA [25].

Among pediatric patients with SCA, stroke is considered to be a major cause of morbidity. Around 11% of these individuals suffer from it during the first two decades of their lives, with higher risk observed during the first decade, especially between the ages of two and five years [26]. Ischemic stroke is more commonly seen among this age group compared to hemorrhagic stroke [26]. Previous transient ischemic attacks, low Hb concentration, recent episodes of ACS, and elevated SBP are considered risk factors for developing it [26]. In this study, 16 cases had ischemic strokes. This prevalence is higher compared to the findings of the study done in Jazan, which had only two cases in their sample [25]. Other individuals might suffer from silent strokes that are only discovered on radiological testing consistent with diffuse white matter disease [27]. This type of stroke can result in cognitive deficiencies [27]. AVN is another complication that results from a temporary or permanent blood loss to the bone and its marrow [28]. Up to 50% of patients with SCD suffer from chronic pain due to AVN [28]. In this study, 13 patients were diagnosed with AVN, which is higher than the aforementioned study in Jazan (11 patients) [25].

As mentioned earlier, due to the loss of the spleen among patients with SCA, infections with encapsulated organisms are commonly seen. Moreover, *S. pneumoniae *is known to be one of the common organisms that cause meningitis [19]. Four patients had meningitis in our sample. Although no recent paper has discussed the incidence of meningitis among children with SCA, the previous study conducted in Makkah found nine cases that suffered from non-specified infections [5].

In the long term, the chronic hemolysis among these patients would result in continuous bilirubin production, which may result in calcium bilirubinate, which would cause gallstones among them [29]. In our study, only four cases were found to develop gallstones. According to a study conducted in 2014, gallstones were more prevalent among SCA patients between 11 and 29 years of age [29]. Early diagnosis and appropriate management can improve their quality of life and increase their survival rates [30]. In general, acute cholecystitis can be managed conservatively; yet, in cases of surgical intervention, a laparoscopic approach is preferable for these patients [30].

As is the case with hospital-based retrospective studies in general, a generalization of our findings is not possible since it only involved pediatrics patients admitted at a single center (KAUH). The fact that only patients in the pediatric age group were included caused our study to have an even smaller sample. However, our observations and findings related to risk factors and complications have been validated by similar findings in previously published studies.

## Conclusions

This study highlights the burden that SCA causes in this region as a major cause of morbidity. It is crucial to screen for hemoglobinopathies to determine the presence of abnormal Hb and its relative levels to diagnose Hb disorders. Screening for newborns can help in the early detection and management of these blood disorders. Also, it provides essential information on carrier status. Additionally, it would also help to limit the occurrence of these inherited disorders and the marriages between those who are carriers or those who are known to suffer from an inherited blood disorder. We recommend that quality medical care and appropriate precautionary measures be offered to these children to promote their health, growth, and development.
